# Impact of Pre-Liver Transplant Treatments on the Imaging Accuracy of HCC Staging and Their Influence on Outcomes

**DOI:** 10.3390/cancers16051043

**Published:** 2024-03-04

**Authors:** Eloisa Franchi, Daniele Eliseo Dondossola, Giulia Maria Francesca Marini, Massimo Iavarone, Luca Del Prete, Clara Di Benedetto, Maria Francesca Donato, Barbara Antonelli, Pietro Lampertico, Lucio Caccamo

**Affiliations:** 1 General and Liver Transplant Surgery Unit, Fondazione IRCCS Ca’ Granda Ospedale Maggiore Policlinico, 20122 Milan, Italy; eloisa.franchi@policlinico.mi.it (E.F.); giulia.marini@unimi.it (G.M.F.M.); luca.delprete@policlinico.mi.it (L.D.P.); barbara.antonelli@policlinico.mi.it (B.A.); lucio.caccamo@policlinico.mi.it (L.C.); 2 Department of Pathophysiology and Transplantation, Università degli Studi, 20122 Milan, Italy; pietro.lampertico@unimi.it; 3 Division of Gastroenterology and Hepatology, Fondazione IRCCS Ca’ Granda Ospedale Maggiore Policlinico, 20122 Milan, Italy; massimo.iavarone@policlinico.mi.it (M.I.); clara.dibenedetto@policlinico.mi.it (C.D.B.); francesca.donato@policlinico.mi.it (M.F.D.)

**Keywords:** liver transplant, hepatocarcinoma, concordance

## Abstract

**Simple Summary:**

Liver transplantation is the gold standard treatment for hepatocarcinoma, and its outcome is strongly influenced by HCC staging. However, concordance between pre-LT radiological and definitive pathological staging remains controversial. The aim of our retrospective study was to assess concordance between radiology and pathology and to explore the factors associated with poor concordance and outcomes. We analyzed all LTs with an HCC diagnosis performed between 2013 and 2018. Concordance (Co group) was defined as a comparable tumor burden in preoperative imaging and post-transplant pathology; otherwise, non-concordance was diagnosed (nCo group). We confirmed the low concordance rate between the radiology and pathology staging systems. The concordance rate between the pre-LT imaging and histopathological results was lower in patients with a high number of nodules. Multiple bridging therapies reduce the accuracy of pre-LT imaging in predicting HCC stages and negatively affect outcomes after LT.

**Abstract:**

The outcome of liver transplantation (LT) for hepatocarcinoma (HCC) is strongly influenced by HCC staging, which is based on radiological examinations in a pre-LT setting; concordance between pre-LT radiological and definitive pathological staging remains controversial. To address this issue, we retrospectively analyzed our LT series to assess concordance between radiology and pathology and to explore the factors associated with poor concordance and outcomes. We included all LTs with an HCC diagnosis performed between 2013 and 2018. Concordance (Co group) was defined as a comparable tumor burden in preoperative imaging and post-transplant pathology; otherwise, non-concordance was diagnosed (nCo group). Concordance between radiology and pathology was observed in 32/134 patients (Co group, 24%). The number and diameter of the nodules were higher when nCo was diagnosed, as was the number of pre-LT treatments. Although concordance did not affect survival, more than three pre-LT treatments led to a lower disease-free survival. Patients who met the Milan Criteria (Milan-in patients) were more likely to receive ≥three prior treatments, leading to a lower survival in multi-treated Milan-in patients than in other Milan-in patients. In conclusion, the concordance rate between the pre-LT imaging and histopathological results was low in patients with a high number of nodules. Multiple bridging therapies reduce the accuracy of pre-LT imaging in predicting HCC stages and negatively affect outcomes after LT.

## 1. Introduction

Liver transplantation (LT) has long been recognized as the best curative treatment for patients with hepatocellular carcinoma (HCC), enabling the simultaneous treatment of either the underlying liver disease or the primary malignant tumor [1]. With a low rate of HCC recurrence (10–14%) and careful patient selection, LT has progressively achieved long-term oncologic outcomes [2]. The Milan Criteria (MC) were the first well-recognized criteria applied to select patients affected by HCC for LT. Since their introduction, imaging techniques have played a central role in both HCC staging and decision making. In fact, the success rates of LT as a curative treatment are attributed to selective listing criteria based on morphological and biological criteria, which have only been introduced in recent years. However, it is well known that radiological staging does not always correspond to subsequent pathologic staging, with significant variations in both the size and number of nodules [3].

Although the MC helped identify patients at low risk of post-transplant HCC recurrence, they potentially precluded access to LT in some patients with a potentially good outcome who could gain the best benefits, and several groups have investigated how these criteria could be expanded without affecting patient survival and tumor recurrence. Therefore, in recent years, the concept of downstaging therapies has emerged, referring to the process of applying locoregional treatments (LRTs) to HCCs currently not meeting the MC at presentation, with the aim of reducing the tumor burden and selecting appropriate candidates for LT [4,5,6,7]. Downstaging provides a viable alternative approach for expanding the limits of the MC and selecting a subgroup of patients whose LT candidacy would otherwise be disregarded. Consequently, the evaluation of patients on the waiting list has changed to a dynamic approach, making the role of imaging increasingly central to transplantation practice. Conversely, these pre-LT treatments have increased the complexity of pre-transplant radiological analysis, and significant differences were found between pre-transplant radiological evaluation and pathological stages of the explanted organ.

In this study, we reviewed all available imaging data of patients from the diagnosis of HCC to LT in a cohort of patients effectively transplanted at our Center for HCC on cirrhosis. We compared the latest available radiological staging with that obtained from pathology in terms of the tumor burden at LT.

## 2. Materials and Methods

This was a retrospective, single-center study of all consecutive patients who underwent LT between 2013 and 2018 at the Fondazione IRCCS Ca’ Granda Ospedale Maggiore Policlinico in Milan, Italy. The inclusion criteria were (i) age > 18 years; (ii) first LT (single or combined) with whole or split cadaver grafts; (iii) indication for transplantation for HCC (MELD < 15) or for HCC associated with primary liver disease with MELD > 15; (iv) previous history of HCC even if there was a complete radiological response at the time of last available imaging before LT; and (v) diagnosis of new-onset HCC on native liver pathology in the absence of a previous history of HCC.

The primary aim was to assess the concordance between imaging and pathology in order to evaluate its ability to predict pre-LT HCC staging.

The secondary endpoints were (1) identification of factors influencing concordance through a detailed characterization of the history of HCC and (2) predictors of post-LT outcomes.

### 2.1. Study Design

**Figure 1:**
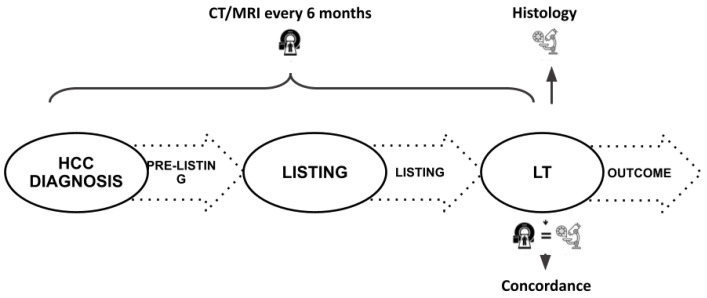
Schematic representation of the study timeline; LT: liver transplant.

Three main time points were identified during the LT pathway: (I) HCC diagnosis, (II) LT listing, and (II) LT procedure. Therefore, in our study, four LT-related timeframes were identified, as shown in Figure 1: the tumor burden (sum of the diameter of the vital nodules) was assessed at HCC diagnosis, and listing and LT were assessed through contrast-enhanced computed tomography (CT) or magnetic resonance (MR) [8]. At LT, a histological examination of the native liver was performed and the pathological tumor burden was evaluated.

The following data were collected from the CT or MR images: the total number of nodules, the sum of the diameters of individual nodules, the diameter of the largest nodule, the total number of active nodules, the sum of the diameters of individual active nodules, the diameter of the largest active nodule, the percentage of necrosis, the presence of macrovascular invasion, and satellite nodules.

Based on the pathological reports, the following parameters were collected: the total number of nodules, the sum of the diameters of individual nodules, the diameter of the largest nodule, the total number of active nodules, the diameter of the major active nodule, the total diameter of the active disease, the diameter of the active portion of the major active nodule, the percentage of necrosis, the presence of micro-and macrovascular invasion, satellite nodules, and foci of mixed hepato-cholangiocellular carcinoma. The TNM classification (TNM stage VIII, Ed. 2017), the stage according to both the Milan and Up-to-Seven Criteria, and the Metroticket score were calculated at all study time points.

Concordance was defined as a comparable tumor burden (ratio between the sum of the diameter of the vital nodules at last CT and histology >70% and <140%) at the last pre-LT imaging and pathology of explanted liver, whereas differences between the results of these two diagnostic approaches led to the definition of discordance. In addition, once HCC staging differed between CT and histology, a definition of discordance was acquired independently from the ratioTBS. In the latter case, understaging or overstaging was considered if the pathology revealed a higher or lower tumor burden than imaging, respectively.

Recipient characteristics and blood tests results were collected from patients’ folders and used to calculate MELD [9], Metroticket [7], and Metroticket 2.0 [10] scores throughout the study period. 

Permission to conduct the study was obtained according to the local institutional board rules and, due to the retrospective nature of the study, the need for written informed consent was waived.

### 2.2. Patients Management

HCC surveillance in patients with chronic liver disease was carried out using abdominal ultrasonography every 6 months according to the guidelines, and either the CT or MR method, or both, was used in the recall policy if a nodule was detected. Whenever imaging techniques were unable to define the nature of the lesion according to the EASL and LiRADS criteria, a liver biopsy for histological diagnosis was performed. Based on the extent of the disease, HCC was classified according to the MC and Up-To-Seven Criteria. The indication for LT was discussed through a multidisciplinary meeting if the patient exceeded a MELD score of 14 and if HCC was not otherwise treatable, according to local and current policies.

After LT, patients transplanted for HCC were enrolled in a specific HCC recurrence surveillance program. CT scans were performed at the first postoperative month, at three months, and every six months thereafter for the first 5 years and according to the patient’s course and condition in the following years. Treatment of recurrence was performed mainly by surgery or LRTs, as indicated, with or without the administration of tyrosine kinase inhibitors (sorafenib and lenvatinib in first-line treatment) or regorafenib and cabozantinib (second-line treatment). The latter remains the only treatment offered, if any, in cases of synchronous recurrence at multiple sites and untreatable progression. In addition, a shift towards immunosuppression based on mTOR inhibitors was considered.

### 2.3. Technical Details 

LT was performed using the modified piggy-back technique according to Belghiti [11] as the method of choice, while the total caval replacement technique remained as an alternative. A venovenous bypass was not performed.

### 2.4. Downstaging and Bridge Therapies

All treatments performed during the pre-listing time were addressed to achieve a radiological response within MC at listing (downstaging) and to maintain that condition while awaiting transplantation (bridging). Up-to-Seven-out stage, whether for unsuccessful downstaging at listing or by disease progression during the waiting time, was defined as a contraindication for LT. The number, type, and timing of downstaging/bridging treatments performed in each patient were registered. Downstaging/bridging procedures included radiofrequency thermal ablation (RFTA), microwave thermal ablation (MWTA), transarterial chemoembolization (TACE), transarterial radioembolization (TARE), percutaneous ethanol injection (PEI), and liver resection, using both open and minimally invasive techniques. The post-treatment response was classified according to RECIST 1.1 criteria [12].

### 2.5. Statistical Analysis

All data are presented as medians (ranges) for categorical variables and as total or relative percentages for continuous variables. For categorical variables, the chi-squared test was used. For continuous variables, means were compared using one-way ANOVA or *t*-tests preceded by a Levene’s test to evaluate the distribution of the data. Analysis of overall and disease-free survival was performed using a Kaplan–Meier analysis followed by a log-rank test. For group comparisons, a cut-off of three pre-LT treatments was used. This value was interpreted as a change point in the time series of the parameters analyzed, as a value more than three was found to be associated with a modified post-LT outcome. Specifically, this value is the minimum value that can be used to identify statistically significant differences between groups. Statistical significance was set at *p* < 0.05. Statistical analysis was performed using SPSS 25 software (Jandel Corporation, Chicago, IL, USA).

## 3. Results

**Figure 2:**
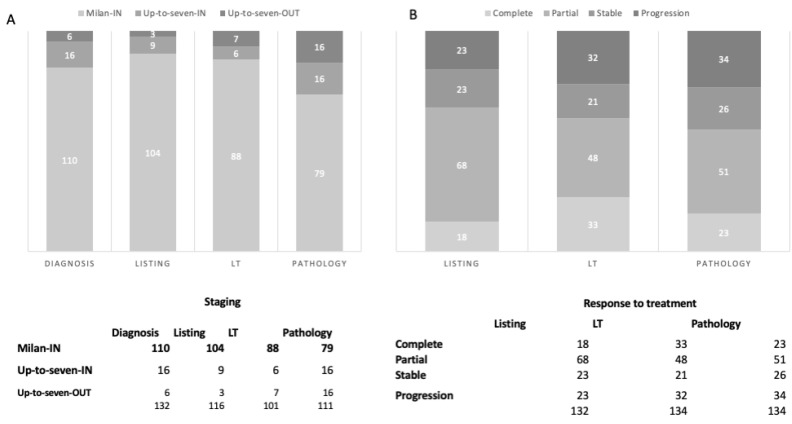
Hepatocellular carcinoma (HCC) staging (**A**) and response to treatment (**B**) during the whole liver transplantation (LT) process. Patients with a complete response or without a HCC diagnosis at a considered timepoint were not included.

At our institution, 293 patients underwent LT during the study period. Of these, 134 patients were diagnosed with HCC and were included in the study (Table 1). The time from HCC first diagnosis to LT was 762.2 (41–5769) days, while the time from diagnosis to enlisting was 621.9 (1–5673) days. The overall follow-up was 1113 (3–2429) days. The primary etiology of liver disease was HCV in 83 (61.9%) patients, HBV in 21 (15.7%) patients, alcohol-related cirrhosis in 16 (11.9%) patients, non-alcoholic steatohepatitis in 8 (6.0%) patients, and other etiologies in 6 (4.5%) patients. The HCC stage during the entire LT pathway (from HCC diagnosis to LT, through downstaging/bridging treatments and radiological re-evaluations) is shown in Figure 2A. HCC was the primary indication for LT in all but two (1.5%) patients who received a diagnosis of HCC once listed for chronic liver failure (and they did not receive downstaging/bridging treatments). At listing, the median AFP level and MELD score were 10 (1–3721) ng/mL and 11 (2–31), respectively, while at LT, the median AFP was 9 (1–60,500) ng/mL and the MELD score was 11 (3–31). Table 1 shows the number and type of treatments performed before LT, and the results of the treatments in the study intervals are reported in Figure 2B. Pathological evaluation of native livers confirmed the presence of active HCC in 111 (46.4%) patients. HCC was single in 30 patients and multiple (number of nodules ranged from 2 to 22) in 81 patients. Among the patients with pT > 2, three showed a maximum nodule diameter of >5 cm (pT3 stage) and two (pT4 stage) an invasion of the diaphragm, radically resected together during the hepatectomy). The pathological HCC stage was reclassified as follows: 79/111 (72%) Milan-in and 16/111 (14%) Up-to-Seven-in (Supplementary Table S1)

HCC recurred after LT in 18 (13%) patients after a median of 479 (96–2140) days. Twenty-five (19%) recipients died after LT (median time 787 (3–1905) days); in 12 (48%) patients, the reason for death was HCC progression (median time 427 days) (Supplementary Table S2).

### 3.1. Comparison between Imaging and Pathology

**Figure 3:**
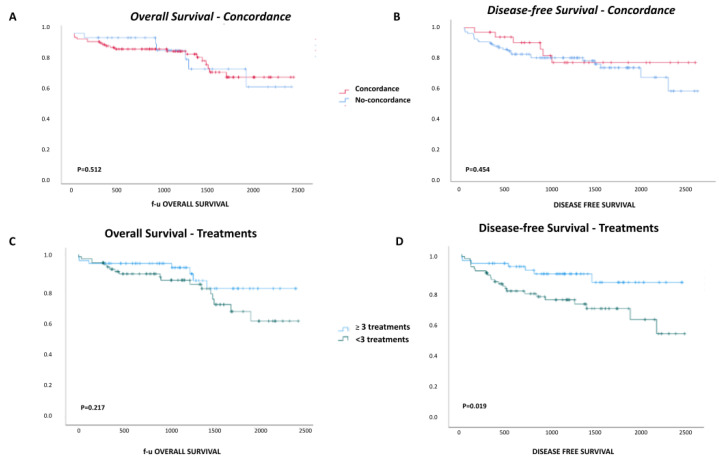
Overall and disease-free survival. Overall (**A**) and disease-free (**B**) survival of patients with a concordant or no tumor burden in imaging and pathological analyses. Overall (**C**) and disease-free (**D**) survival of patients that received less than or three or more pre-liver transplantation hepatocellular carcinoma treatments.

Concordance between radiology and pathology was found in 32/134 (Co group, 24%; ratioTBS 87 (73–135)) patients, while in the other 102/134 (nCo group, 76%; ratioTBS 55 (10–223)), the last pre-LT imaging and pathology reports were discordant. Table 2 shows the comparison between the Co and nCo groups. Radiological examination revealed a significant underestimation of several parameters. In particular, the number of nodules, total diameter of nodules, number of viable nodules, and total diameter of viable nodules were significantly higher in patients with nCo. Satellitosis was more frequently detected in the nCo group, as well as in the time from HCC diagnosis to LT, even if they did not reach statistical significance. However, both 1–3–5-year overall and disease-free survival did not differ between the Co and no-Co groups (Figure 3A,B).

### 3.2. Role of Downstaging/Bridging Procedures

Our patients received a mean of 2.2 (0–8) treatments before LT. The number of pre-LT procedures was significantly higher in the nCo group than in the Co patients (2.5 (0.0–8.8) vs. 1.7 (0.0–8.1), *p* = 0.039). As pre-LT treatments represent a key issue in HCC management during listing, and their impact on imaging and results is still debated, we performed a group comparison based on the number of treatments. Among patients who received up to two (<three treatments) pre-LT HCC treatments, 36 (65.5%) belonged to the nCo group and 19 (34.5%) to the Co group (*p* = 0.23), and among those who received at least three treatments (≥three treatments), 66 (84%) were classified as nCo and 13 (16%) as Co (*p* = 0.014) (Figure 2). In patients who received ≥three treatments, pathology showed a higher number of nodules and a larger total diameter of both the overall (*p* = 0.04 and *p* = 0.001, respectively) and active nodules (*p* = 0.019, *p* = 0.026, respectively) compared to patients with less than three treatments before LT. Interestingly, the time from the last treatment to LT was shorter when <three treatments were performed (*p* < 0.001). Disease-free survival at 1–3–5 years was lower in patients receiving ≥three treatments (83%, 72%, and 67% vs. 95%, 72%, and 67%, *p* = 0.019), while overall survival did not differ between the two groups of patients (Figure 3C,D).

### 3.3. Impact of HCC Staging 

Among the 102 patients showing a Milan-in stage at pathology, 48% (49/102) received less than three pre-LT neoadjuvant treatments, of which 6/102 (6%) received no treatment at all. Moreover, ≥three treatments were more frequently found in Milan-in patients at pathology than in Milan-out patients (*p* = 0.011). Both overall and disease-free survival rates were higher in the Milan-in and complete response patients at pathology (*n* = 102) than in the 32 Milan-out patients (*p* = 0.013) (Supplementary Figure S2). Once the analysis was restricted to Milan-in and complete response patients, the disease-free survival of those who received <three treatments (55 patients) was superior to those who received ≥three treatments (47 patients) (*p* = 0.019) (Supplementary Figure S3). Conversely, the survival of these latter patients did not differ from that of the Milan-out patients (Supplementary Figure S3).

## 4. Discussion

In recent years, due to the expansion of HCC inclusion criteria for liver transplantation, the number of pre-transplant treatments with downstaging intent has progressively increased, and the role of imaging has grown in parallel. Therefore, we aimed to evaluate whether pre-LT imaging techniques are reliable in predicting the stage of disease in accordance with pathological findings to explore the eventual role of multiple pre-LT treatments of HCC in patient survival. Through our analyses, we showed the limits of HCC staging before LT, obtained using imaging techniques, especially in patients with a higher tumor burden. Our results show that, although this limitation does not impact outcomes, a high number of pre-TL treatments is correlated with a lower disease-free survival. In addition, an increased requirement for pre-transplant treatments, coupled with a diminished outcome, suggests a more advanced initial condition for patients necessitating more than three bridging treatments or patients with a more aggressive tumor biology.

Liver transplantation is the best treatment for HCC because of its superior oncologic outcomes compared to liver resection and LRTs and its unique ability to cure underlying liver disease [4]. However, LT improved oncological outcomes as a direct consequence of careful patient selection based on the patients’ general conditions and tumor staging. The current evaluation of transplantability of HCC patients is based on morphological criteria, with AFP as the only surrogate of tumor biology, while the number of treatments needed to obtain the downstaging and the duration of response are used as a surrogate of tumor behavior [13]. Thus, radiological investigations and their findings, such as the number and size of tumor nodules and tumor enhancement, are indispensable foundations of this strategy. The reliability of radiological data compared to the results of pathological examination of the native liver after LT is crucial for obtaining the best possible outcome for the recipients. However, numerous studies have pointed out that this concordance is rarely achieved [14,15,16,17,18]. Our results showed a concordance rate of 24%, thus confirming the low accuracy of imaging techniques in pre-LT staging [19]. In a recent review of the literature, the sensitivity of CT for HCC detection of any size and stage was 77.5% (95% CI, 70.9–82.9%) and the specificity was 91.3% (95% CI, 86.5–94.5%) [20]. Despite different studies emphasizing the superior sensitivity of MR over CT in this setting [21,22,23], Krinsky et al. showed that MR inadequately detects tumors of between one and three centimeters (concordance rate 52%) and less than one centimeter (concordance rate 4%) [24]. Different factors could be responsible for these results; in our study, the size and number of nodules (both total and viable) and the number of treatments were identified as factors impairing concordance [25,26]. In addition, as suggested by our data, the low reliability of detecting small tumors in cirrhotic livers, the inaccurate discrimination between tumor and regenerative nodules, and tumor persistence/recurrence after treatments could amplify the limitations of MR and CT accuracy [17]. As HCC understaging plays a significant role in the decision on the transplantability of patients, the integration of more sensitive diagnostic modalities should be investigated to achieve a better selection of patients. 

The number of pre-transplant treatments also appears to be crucial in determining the concordance between imaging and pathology [16]. In our study, the number of downstaging and bridging treatments was significantly different between the two groups analyzed. Indeed, according to the number of treatments, the concordance was 34% in patients who underwent < three treatments and only 16% in those who underwent ≥three treatments. While these data are consistent with those already published in the literature [27], it is clear that a patient with a multifocal tumor at presentation and with a poor response to LRTs will need multiple treatments to fall within or remain within the Milan criteria. In our study, the Milan-in stage at LT was more often achieved through a long and complex downstaging process. Collectively, Milan-in patients showed better disease-free and overall survival than Milan-out patients. However, the need for multiple treatments from presentation to LT to reach or maintain the Milan-in stage does not differ significantly from that of Milan-out patients. Once again, we show that three is the changing point number for downstaging and bridging treatments; the real usefulness of going beyond three treatments to control tumor burden before LT to improve post-LT survival has yet to be demonstrated. Our data support the evidence that a history of multiple pre-LT treatments negatively affects the post-LT course. Indeed, in a recent study by Shimada et al. [28], a statistically significant relationship between multiple pre-LT treatments and hepatocarcinoma recurrence was reported (although a cut-off for the number of treatments was not identified). It should be noted that despite the reduction in post-LT outcomes, patient survival is far above the 50% 5-year cutoff used to justify LT for oncological indications.

Despite data regarding the impact of the number of bridging/downstaging treatments on both staging and post-LT outcomes, this factor is still not present in the most commonly used HCC scores. Although some of them (e.g., the SOFT score [29]) include previous surgery or the response to treatment as a variable, the need for repeated locoregional treatments has not been yet included. Cucchetti et al. [30] suggested the inclusion of the response to neoadjuvant therapies in Metroticket 2.0. In this study, they showed that the modified Metroticket can be used to better evaluate LT eligibility and predict patient outcomes. However, the low sensitivity of imaging techniques in detecting post-treatment responses could affect their efficacy [10]. Our analysis further emphasizes the importance of the number of pre-LT treatments to better and more accurately depict patients’ staging and, consequently, their outcomes. In an effort to predict HCC recurrence and based on the different scores based on pathological findings [7,31,32,33], the number of locoregional treatments before LT could be used as a surrogate for tumor aggressiveness and/or advanced HCC at presentation, such as AFP [34,35,36,37]. 

Our study has several limitations. First, the number of patients included was relatively small and they were evaluated retrospectively. Although the single-center nature of our study is the main cause of these limitations, the ability to thoroughly evaluate the patient’s history from HCC diagnosis to transplantation allowed us to include a large number of variables and identify the treatment cut-off. Although the concordance of HCC was evaluated only using CT in our series, the adoption of MRI as a pre-LT imaging technique could improve HCC staging, and its possible impact on post-LT results should be evaluated.

## 5. Conclusions

Our study confirmed the low concordance rate between the radiology and pathology staging systems. This issue strongly affects the selection of patients and their eligibility for transplantation, as well as their post-transplant outcomes. A high tumor burden decreases the concordance rate between the pre-LT imaging and histopathological findings. In the era of expanded HCC criteria, multiple bridging treatments decrease the accuracy of pre-LT imaging in predicting HCC stages and adversely affect the outcomes of transplantation. Future research should address these shortcomings and consider a path toward a more unified radiologic–pathologic staging system that can provide more precise and accurate information about a patient’s pathological status.

## Figures and Tables

**Table 1:** Overall characteristics of the study population.

	Patients (*n* = 134)
FEATURES	
Age, years	59 (36–71)
Gender, M, *n* (%)	113 (84.3)
HCV RNA positive, *n* (%)	29 (21.6)
Donor age, years	64 (17–88)
N° (%) of patients treated at least one time with:	
- TACE	95 (70.9)
- TARE	1 (0.7)
- RFTA/MWTA	53 (39.6)
- Liver resection, open	11 (8.2)
- Liver resection, laparoscopic	6 (4.5)
- PEI	7 (5.2)
AT LISTING	
AFP, ng/mL	10 (1–3721)
MELD	11 (2–31)
HCC, *n* (%)	132 (98.5)
Time from diagnosis to listing, days	379 (−74–5673)
AT LIVER TRANSPLANTATION	
AFP, ng/mL	9 (1–60500)
MELD	11 (3–31)
Imaging, *n* (%)	
- TC	123 (91.8)
- RM	11 (8.2)
HCC, *n* (%)	101 (75.4)
Active nodules, *n*	2 (1–7)
Larger active nodule, diameter, mm	16 (4–45)
Active nodules, total diameter, mm	25 (4–108)
Macrovascular invasion, *n* (%) *	1 (1.0)
Metroticket 2.0	94.1 (42.4–97.6)
Last imaging–LT interval, days	52 (1–154)
Presentation–LT interval, days	499.5 (41–5769)
Waiting time, days	73 (2–1328)
AT PATHOLOGICAL EXAMINATION	
HCC nodules, *n.* (%)	111 (82.8)
Active nodules, *n.*	2 (1–22)
Larger active nodule, diameter, mm	22 (6–78)
Actrive nodules, total diameter, mm	38 (8–239)
Microvascular invasion, *n* (%) °	29 (26.1)
Edmondson grade, *n* (%) °	
- 1	7 (6.3)
- 2	32 (28.8)
- 3	66 (59.5)
- 4	6 (5.4)
Metroticket original	72.1 (41.1–78.4)

* Percentage is intended to be calculated on total patients with HCC at imaging (*n* = 101). ° The percentage is intended to be calculated using the total number of patients with histology-positive HCC in native liver (*n* = 111).

**Table 2:** Overall characteristics of the concordant and non-concordant cases in the study population. Concordance was defined as comparable tumor burden at pre-LT imaging and pathology, whereas differences between the two diagnostic techniques led to the definition of discordance.

	Concordant (*n* = 32)	Non Concordant (*n* = 102)	*p* Value
Age, years	59.5 (49–71)	59 (36–71)	0.581
Gender, M, *n* (%)	26 (81.2)	87 (85.3)	0.584
MELD at listing	11 (6–26)	11 (2–31)	0.297
AFP level at listing, ng/mL	9 (1–864)	11.5 (1–3721)	0.581
MELD at LT	10 (6–30)	11 (3–31)	0.899
AFP at LT, ng/mL	9.5 (2–755)	9 (1–60500)	0.581
Donor age, years	60 (18–80)	65 (17–88)	0.074
Waiting time, days	73 (6–1328)	73 (2–1170)	0.512
LT–HCC recurrence interval, days	510 (107–820)	360 (96–2124)	0.835
Last imaging–LT interval, days	54 (12–154)	50 (1–147)	0.308
Treatment ≥ 3, *n* (%)	13 (40.6)	66 (64.7)	0.014
Everolimus, *n* (%)	4 (12.5)	24 (23.5)	0.107
HCC recurrence, *n* (%)	5 (15.6)	13 (12.7)	0.437
Time from HCC diagnosis to LT, days	455.5 (63–1572)	591.5 (4–5769)	0.062
Overall patients’ survival, *n* (%)			
- 1 year	31 (96.9)	93 (91.1)	0.945
- 3 years	29 (87.6)	90 (87.3)	
- 5 years	27 (74.4)	83 (69.2)	
Disease-free survival, *n* (%)			
- 1 year	30 (93.5)	88 (86.2)	0.454
- 3 years	26 (76.1)	82 (79.3)	
- 5 years	26 (76.1)	79 (72.5)	
AT PATHOLOGICAL EXAMINATION			
Nodules, *n*.	2 (1–4)	3 (1–29)	0.004
Nodules, total diameter, mm	35 (8–61)	59 (8–271)	0.001
Large nodule, diameter, mm	22 (8–45)	25 (8–78)	0.066
Active nodules, *n*.	2 (1–3)	2 (1–22)	0.019
Active nodules, diameter, mm	32 (8–61)	40.5 (8–239)	0.026
Larger active nodule, diameter, mm	21 (8–45)	22.5 (6–78)	0.348
Active nodules, total diameter, mm	23 (3–34)	26 (1–78)	0.097
Metroticket original, %	73.8 (48.7–78.4)	70.4 (41.1–78.4)	0.081
TNM, *n* (%) **			
- ≤1	10 (37.0)	20 (23.8)	0.108
- >1	17 (63.0)	64 (76.2)	
Edmondson grade, *n* (%) **			
- ≤2	7 (25.9)	32 (38.1)	0.149
- >2	20 (74.1)	52 (61.9)	
Microsatellite, *n* (%)	2 (6.3)	19 (18.6)	0.054
Microvascular invasion, *n* (%)	6 (18.8)	23 (22.5)	0.427
Staging, *n* (%)			
- Milan-in	31 (96.9)	71 (69.6)	0.002
- Up-to-Seven-in	1 (3.1)	15 (14.7)	
- Up-to-Seven-out	0 (0.0)	16 (15.7)	

** Percentages are intended to be calculated on patients with histology-positive HCC on native liver (*n* = 111, Co = 27, nCo = 84).

## Data Availability

The data presented in this study are available on request from the corresponding author. The data are not publicly available due to privacy reasons.

## Supplementary Materials

The following supporting information can be downloaded at: https://www.mdpi.com/article/10.3390/cancers16051043/s1, Supplementary Table S1: Classification according to the Milan Criteria; Supplementary Table S2: HCC status at transplant; Supplementary Figure S1: Overall and disease-free survival of patients with Milan-in and Milan-out hepatocellular carcinoma staging in pathological analyses; Supplementary Figure S2: Overall and disease-free survival of patients with Milan-in hepatocellular carcinoma staging in pathological analyses achieved with a number of pre-liver transplant treatments inferior or equal/superior to three; Supplementary Figure S3: Overall and disease-free survival of patients with Milan-in hepatocellular carcinoma staging in pathological analyses achieved with a number of pre-liver transplantation equal/superior to three and Milan-out patients.

## References

[1] Adam R., Karam V., Cailliez V., Grady J.G.O., Mirza D., Cherqui D., Klempnauer J., Salizzoni M., Pratschke J., Jamieson N. (2018). 2018 Annual Report of the European Liver Transplant Registry (ELTR)—50-Year Evolution of Liver Transplantation. Transpl. Int..

[2] Belghiti J., Fuks D. (2012). Liver Resection and Transplantation in Hepatocellular Carcinoma. Liver Cancer.

[3] Cunha G.M., Hosseini M., Furlan A., Fowler K.J. (2022). Hepatocellular Carcinoma Staging: Differences Between Radiologic and Pathologic Systems and Relevance to Patient Selection and Outcomes in Liver Transplantation. AJR Am. J. Roentgenol..

[4] Yao F.Y., Ferrell L., Bass N.M., Bacchetti P., Ascher N.L., Roberts J.P. (2002). Liver Transplantation for Hepatocellular Carcinoma: Comparison of the Proposed UCSF Criteria with the Milan Criteria and the Pittsburgh Modified TNM Criteria. Liver Transplant..

[5] Llovet J.M., Pavel M., Rimola J., Diaz M.A., Colmenero J., Saavedra-Perez D., Fondevila C., Ayuso C., Fuster J., Ginès P. (2018). Pilot Study of Living Donor Liver Transplantation for Patients with Hepatocellular Carcinoma Exceeding Milan Criteria (Barcelona Clinic Liver Cancer Extended Criteria). Liver Transplant..

[6] Halazun K.J., Sapisochin G., von Ahrens D., Agopian V.G., Tabrizian P. (2020). Predictors of Outcome after Liver Transplantation for Hepatocellular Carcinoma (HCC) beyond Milan Criteria. Int. J. Surg..

[7] Mazzaferro V., Llovet J.M., Miceli R., Bhoori S., Schiavo M., Mariani L., Camerini T., Roayaie S., Schwartz M.E., Grazi G.L. (2009). Predicting Survival after Liver Transplantation in Patients with Hepatocellular Carcinoma beyond the Milan Criteria: A Retrospective, Exploratory Analysis. Lancet Oncol..

[8] Lencioni R., Llovet J.M. (2010). Modified RECIST (mRECIST) Assessment for Hepatocellular Carcinoma. Semin. Liver Dis..

[9] Kamath P.S., Kim W.R. (2007). The Model for End-Stage Liver Disease (MELD). Hepatology.

[10] Mazzaferro V., Sposito C., Zhou J., Pinna A.D., De Carlis L., Fan J., Cescon M., Di Sandro S., Yi-Feng H., Lauterio A. (2018). Metroticket 2.0 Model for Analysis of Competing Risks of Death After Liver Transplantation for Hepatocellular Carcinoma. Gastroenterology.

[11] Belghiti J., Panis Y., Sauvanet A., Gayet B., Fékété F. (1992). A New Technique of Side to Side Caval Anastomosis during Orthotopic Hepatic Transplantation without Inferior Vena Caval Occlusion. Surg. Gynecol. Obstet..

[12] Eisenhauer E.A., Verweij J. (2009). 11 New Response Evaluation Criteria in Solid Tumors: RECIST GUIDELINE VERSION 1.1. EJC Suppl..

[13] Ruch B., Wagler J., Kumm K., Zhang C., Katariya N.N., Garcia-Saenz-de-Sicilia M., Giorgakis E., Mathur A.K. (2022). Hepatocellular Carcinoma, Alpha Fetoprotein, and Liver Allocation for Transplantation: Past, Present and Future. Curr. Oncol..

[14] Sotiropoulos G.C., Paul A., Molmenti E., Lang H., Frilling A., Napieralski B.P., Nadalin S., Treckmann J., Brokalaki E.I., Gerling T. (2005). Liver Transplantation for Hepatocellular Carcinoma in Cirrhosis within the Eurotransplant Area: An Additional Option with “Livers That Nobody Wants”. Transplantation.

[15] Tovoli F., Renzulli M., Negrini G., Brocchi S., Ferrarini A., Andreone A., Benevento F., Golfieri R., Morselli-Labate A.M., Mastroroberto M. (2018). Inter-Operator Variability and Source of Errors in Tumour Response Assessment for Hepatocellular Carcinoma Treated with Sorafenib. Eur. Radiol..

[16] Vicentin I., Mosconi C., Garanzini E., Sposito C., Serenari M., Buscemi V., Verna M., Spreafico C., Golfieri R., Mazzaferro V. (2021). Inter-Center Agreement of mRECIST in Transplanted Patients for Hepatocellular Carcinoma. Eur. Radiol..

[17] Ecker B.L., Hoteit M.A., Forde K.A., Hsu C.C., Reddy K.R., Furth E.E., Siegelman E.S., Habibollahi P., Ben-Josef E., Porrett P.M. (2018). Patterns of Discordance Between Pretransplant Imaging Stage of Hepatocellular Carcinoma and Posttransplant Pathologic Stage: A Contemporary Appraisal of the Milan Criteria. Transplantation.

[18] Vigano L., Terrone A., Costa G., Franchi E., Cimino M., Procopio F., Del Fabbro D., Torzilli G. (2022). Effect of Chemotherapy on Tumour-Vessel Relationship in Colorectal Liver Metastases. Br. J. Surg..

[19] Lee Cheah Y., Chow P.K.H. (2012). Liver Transplantation for Hepatocellular Carcinoma: An Appraisal of Current Controversies. Liver Cancer.

[20] Nadarevic T., Giljaca V., Colli A., Fraquelli M., Casazza G., Miletic D., Štimac D. (2021). Computed Tomography for the Diagnosis of Hepatocellular Carcinoma in Adults with Chronic Liver Disease. Cochrane Database Syst. Rev..

[21] Roberts L.R., Sirlin C.B., Zaiem F., Almasri J., Prokop L.J., Heimbach J.K., Murad M.H., Mohammed K. (2018). Imaging for the Diagnosis of Hepatocellular Carcinoma: A Systematic Review and Meta-Analysis. Hepatology.

[22] Moura Cunha G., Chernyak V., Fowler K.J., Sirlin C.B. (2021). Up-to-Date Role of CT/MRI LI-RADS in Hepatocellular Carcinoma. J. Hepatocell. Carcinoma.

[23] Ayuso C., Rimola J., Vilana R., Burrel M., Darnell A., García-Criado Á., Bianchi L., Belmonte E., Caparroz C., Barrufet M. (2018). Diagnosis and Staging of Hepatocellular Carcinoma (HCC): Current Guidelines. Eur. J. Radiol..

[24] Krinsky G.A., Lee V.S., Theise N.D., Weinreb J.C., Morgan G.R., Diflo T., John D., Teperman L.W., Goldenberg A.S. (2002). Transplantation for Hepatocellular Carcinoma and Cirrhosis: Sensitivity of Magnetic Resonance Imaging. Liver Transplant..

[25] Grąt K., Grąt M., Rowiński O., Patkowski W., Zieniewicz K., Pacho R. (2018). Accuracy of Computed Tomography in the Assessment of Milan Criteria in Liver Transplantation for Hepatocellular Carcinoma. Transplant. Proc..

[26] Jeng K.-S., Huang C.-C., Lin C.-K., Lin C.-C., Huang C.-T., Chung C.-S., Weng M.-T., Chen K.-H. (2019). Reappraisal of Failures in Downstaging Treatment of Hepatocellular Carcinoma Prior to Liver Transplant-Preliminary Report on the Impact of Underestimations of Tumor Numbers and Tumor Sizes as Measured From Imaging Before Transplant. Transplant. Proc..

[27] Agopian V.G., Harlander-Locke M.P., Ruiz R.M., Klintmalm G.B., Senguttuvan S., Florman S.S., Haydel B., Hoteit M., Levine M.H., Lee D.D. (2017). Impact of Pretransplant Bridging Locoregional Therapy for Patients With Hepatocellular Carcinoma Within Milan Criteria Undergoing Liver Transplantation: Analysis of 3601 Patients From the US Multicenter HCC Transplant Consortium. Ann. Surg..

[28] Shimada S., Shamaa T., Ivanics T., Kitajima T., Adhnan M., Collins K., Rizzari M., Yoshida A., Abouljoud M., Salgia R. (2022). Multiple Pretransplant Treatments for Patients Without Pathological Complete Response May Worsen Posttransplant Outcomes in Patients with Hepatocellular Carcinoma. Ann. Surg. Oncol..

[29] Rana A., Hardy M.A., Halazun K.J., Woodland D.C., Ratner L.E., Samstein B., Guarrera J.V., Brown R.S., Emond J.C. (2008). Survival Outcomes Following Liver Transplantation (SOFT) Score: A Novel Method to Predict Patient Survival Following Liver Transplantation. Am. J. Transpl..

[30] Cucchetti A., Serenari M., Sposito C., Di Sandro S., Mosconi C., Vicentin I., Garanzini E., Mazzaferro V., De Carlis L., Golfieri R. (2020). Including mRECIST in the Metroticket 2.0 Criteria Improves Prediction of Hepatocellular Carcinoma-Related Death after Liver Transplant. J. Hepatol..

[31] Parfitt J.R., Marotta P., Alghamdi M., Wall W., Khakhar A., Suskin N.G., Quan D., McAllister V., Ghent C., Levstik M. (2007). Recurrent Hepatocellular Carcinoma after Transplantation: Use of a Pathological Score on Explanted Livers to Predict Recurrence. Liver Transplant..

[32] Iwatsuki S., Dvorchik I., Marsh J.W., Madariaga J.R., Carr B., Fung J.J., Starzl T.E. (2000). Liver Transplantation for Hepatocellular Carcinoma: A Proposal of a Prognostic Scoring system11No Competing Interests Declared. J. Am. Coll. Surg..

[33] Decaens T., Roudot-Thoraval F., Badran H., Wolf P., Durand F., Adam R., Boillot O., Vanlemmens C., Gugenheim J., Dharancy S. (2011). Impact of Tumour Differentiation to Select Patients before Liver Transplantation for Hepatocellular Carcinoma. Liver Int..

[34] Mehta N., Heimbach J., Harnois D.M., Sapisochin G., Dodge J.L., Lee D., Burns J.M., Sanchez W., Greig P.D., Grant D.R. (2017). Validation of a Risk Estimation of Tumor Recurrence After Transplant (RETREAT) Score for Hepatocellular Carcinoma Recurrence After Liver Transplant. JAMA Oncol..

[35] Mehta N., Dodge J.L., Roberts J.P., Yao F.Y. (2018). Validation of the Prognostic Power of the RETREAT Score for Hepatocellular Carcinoma Recurrence Using the UNOS Database. Am. J. Transpl..

[36] Mehta N., Yao F.Y. (2019). What Are the Optimal Liver Transplantation Criteria for Hepatocellular Carcinoma?. Clin. Liver Dis..

[37] Costentin C., Piñero F., Degroote H., Notarpaolo A., Boin I.F., Boudjema K., Baccaro C., Podestá L.G., Bachellier P., Ettorre G.M. (2022). R3-AFP Score Is a New Composite Tool to Refine Prediction of Hepatocellular Carcinoma Recurrence after Liver Transplantation. JHEP Rep..

