# Supplementary material for: Impact of Pre-Liver Transplant Treatments on the Imaging Accuracy of HCC Staging and Their Influence on Outcomes
Source: Cancers (Basel). 2024 Mar 4;16(5):1043. doi: 10.3390/cancers16051043 (PMC10930548; doi:10.3390/cancers16051043)
Supplement: Supplementary file 1 [file cancers-16-01043-s001.zip › cancers-2782058-supplementary.pdf]

Table S1. Classification according to the Milan Criteria

	DIAGNOSIS	LISTING	TRANSPLANT	
			Radiology	Pathology
MILAN-IN	110 (83%)	120 (91%)	88	79
UP TO 7 IN	16 (12%)	9 (7%)	7	16
UP TO 7 OUT	6 (5%)	3 (2%)	6	16
**TOTAL**	**132***	**132**	**101**	**111**

* 2 patients with HCC diagnosis at transplant

Table S2. HCC status at transplant

	RADIOLOGY	PATHOLOGY
COMPLETE RESPONSE	18	33
PARTIAL RESPONSE	68	48
STABLE DISEASE	23	21
PROGRESSION DISEASE	23	32
**TOTAL**	**132**	**134**
	RADIOLOGY	PATHOLOGY
T1	41	30
T2	59	76
T3	0	3
T4	1	2
**TOTAL**	**101**	**111**

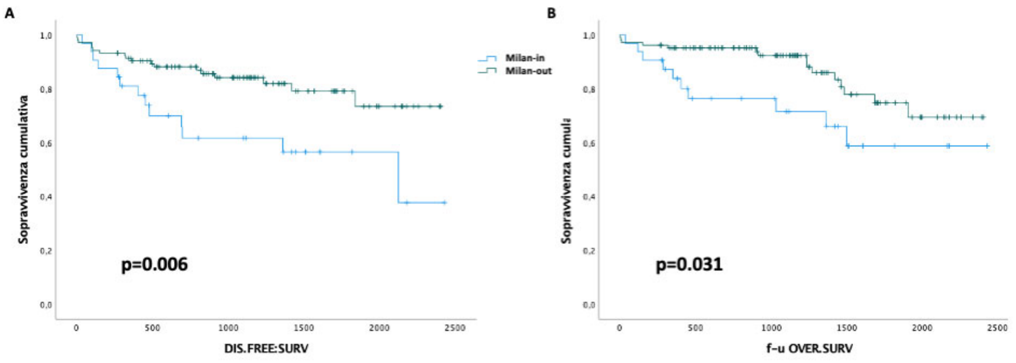

Figure S1. Overall (**A**) and disease free (**B**) survival of patients with Milan-in and Milan-out hepatocellular carcinoma staging at pathological analyses.

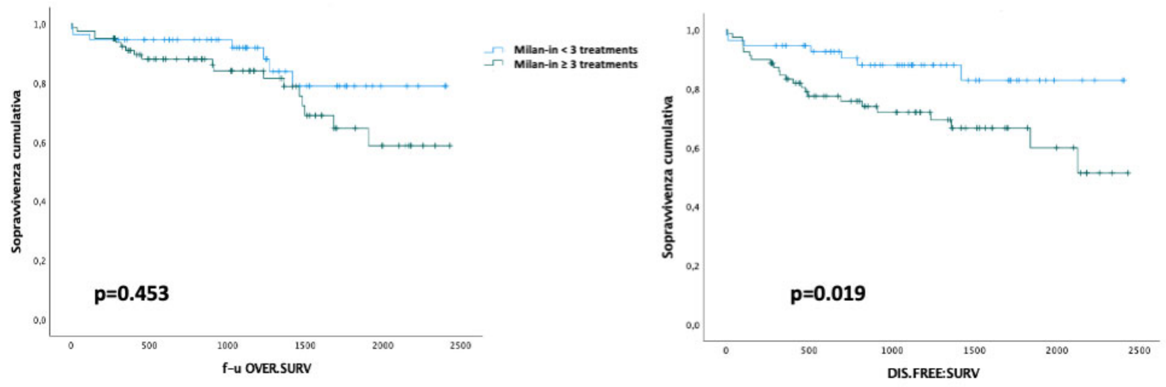

Figure S2. Overall (**A**) and disease free (**B**) survival of patients with Milan-in hepatocellular carcinoma staging at pathological analyses achieved with a number of pre-liver transplantation treatments inferior or equal/superior to 3.

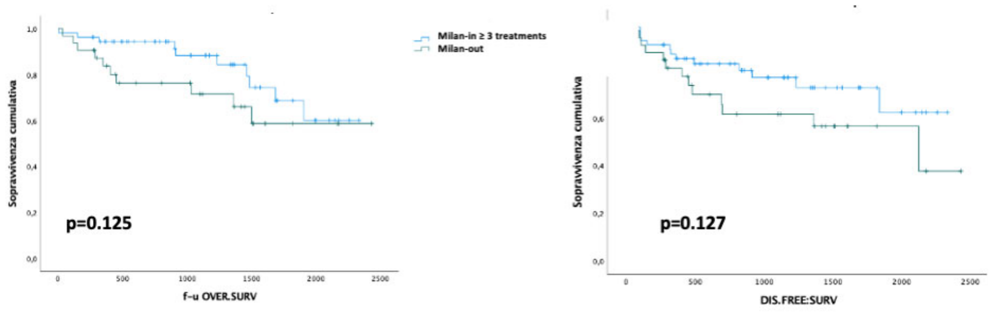

Figure S3. Overall (**A**) and disease free (**B**) survival of patients with Milan-in hepatocellular carcinoma staging at pathological analyses achieved with a number of pre-liver transplantation or equal/superior to 3 and Milan-out patients.
